# Development of an MR-compatible DOI-PET detector module

**DOI:** 10.1186/2197-7364-2-S1-A4

**Published:** 2015-05-18

**Authors:** Qingyang Wei, Shi Wang, Tianpeng Xu, Yunpeng Gao, Yaqiang Liu, Tianyu Ma

**Affiliations:** Department of Electrical Engineering, Tsinghua University, Beijing, China; Department of Engineering Physics, Tsinghua University, Beijing, China; Key Laboratory of Particle and Radiation Imaging, Ministry of Education (Tsinghua University), Beijing, China

Silicon Photomultiplier (SiPM) is a promising sensor for MR-compatible PET systems. In this paper, we developed a compact 2-layer DOI-PET detector. The top layer is a 15Ã—15 LYSO array, and the crystal size is 2x2x7mm^3^. The bottom layer is a 16Ã—16 array with the same size crystals. There is half-crystal offset between two layers in both transverse directions. The detector is coupled to an 8Ã—8 SiPM array (MicroFB-30035-SMT, Sensl). Sixty-four channels of SiPMs are read out by an ASIC chip with in-chip multiplexing resistor networks in the form of two position and one energy analog signals, and are then converted to wave-form digital signals with 80 MHz 12-bit ADC chips. The energy is calculated by averaging the 3 points around the peak of the pulse. Flood images with two 22Na point sources irradiated on the top and at the bottom of the detector module were acquired. The results show that the detector module achieves good crystal identification capability in both layers with an average energy resolution of 17.1% at 511 keV.

